# Electrochemical Study and Determination of Homovanillic Acid, the Final Metabolite of Dopamine, Using an Unmodified Disposable Electrode

**DOI:** 10.3390/molecules30020369

**Published:** 2025-01-17

**Authors:** Mihaela Buleandră, Lavinia Georgiana Voica, Dana Elena Popa, Irinel Adriana Badea, Emilia Elena Iorgulescu, Mihaela Carmen Cheregi

**Affiliations:** Department of Analytical Chemistry and Physical Chemistry, Faculty of Chemistry, University of Bucharest, Panduri Avenue 90-92, District 5, 050663 Bucharest, Romania; lavinia.voica@s.unibuc.ro (L.G.V.); irinel.badea@chimie.unibuc.ro (I.A.B.); emilia-elena.iorgulescu@chimie.unibuc.ro (E.E.I.); mihaela.cheregi@g.unibuc.ro (M.C.C.)

**Keywords:** homovanillic acid, pencil graphite electrode, voltammetry, plasma sample

## Abstract

This paper summarizes the main findings of a study which aimed to examine the electrochemical oxidation of homovanillic acid (HVA), the final metabolite of dopamine. A pencil graphite electrode (PGE) was used as working electrode and the measurements were performed by cyclic voltammetry (CV) and differential pulse voltammetry (DPV). The type and the composition of the graphite leads used as PGE, the pH of the supporting electrolyte, as well as the scan rates were optimized by CV. The analyte was irreversibly oxidized in Britton–Robinson buffer (BRB) solutions. The interpretation of the voltammetric signals and the correlation of the acquired information were the key to addressing the electrode process undergone by HVA at the PGE. The outcomes of the pH and scan rate studies led to the conclusion that two electrons and two protons were involved in the diffusion-controlled process. Using the PGE, a linear relationship between peak current and HVA concentration was obtained between 1.0 × 10^−6^ M and 5.0 × 10^−5^ M by DPV in BRB with pH 2.0. The detection limit of 3.84 × 10^−7^ M was calculated. The accuracy, the precision, and the selectivity of the quantitative method have successfully undergone evaluation. The practical application of the developed voltammetric method was checked by determining the HVA concentration in spiked plasma samples, yielding good recovery values.

## 1. Introduction

Homovanillic acid (HVA, (4-hydroxy-3-methoxyphenyl) acetic acid) is the end product of dopamine (DA) metabolism in the human body, a catecholamine neurotransmitter that plays a vital role in many critical functions, including memory and learning, attention, pleasure and reward, as well as motivation or voluntary movement [1]. DA is metabolized to HVA in a series of reactions by enzymes such as aldehyde dehydrogenase (ALDH), monoamine oxidase (MAO), or catechol-O-methyltransferase (COMT). In one possible pathway, DA is oxidized to 3,4-dihydroxyphenylacetic acid (DOPAC) by MAO and ALDH, and then COMT oxidizes DOPAC to HVA. Alternatively, COMT catalyzes DA to 3-methoxytyramine (3-MT), which is further oxidized to HVA by MAO and ALDH [2].

Dysfunction in dopamine neurotransmission is an indicator of most metabolic, neurological, and psychiatric disorders. Low DA levels can cause Parkinson’s disease (PD), attention deficit hyperactivity disorder (ADHD), depression, and restless leg syndrome [3], while mania, obesity, and addiction are associated with high DA levels [4,5]. Cerebrospinal fluid (CSF) HVA levels are low in untreated PD patients, increase after the initiation of levodopa treatment, and are correlated with impairment of motor activity, so a low concentration of HVA in CSF indicates DA depletion in the brain [6]. Thus, HVA concentrations in biological fluids could be a valuable biomarker for diagnosis and prognosis of some diseases related to DA deficiency such as PD and also for drug dosage optimization and treatment monitoring [7]. HVA is an indicator of the functioning of the dopaminergic pathway in the central nervous system and its concentration in CFS provides an estimation of the dopamine turnover in the mesostriatal and mesolimbic areas [8,9]. For example, an increase in the HVA/DA ratio reflects an increase in striatal DA biosynthesis [10].

Low plasma HVA levels are also associated with tobacco use, alcoholism or with delirium tremens during alcohol withdrawal [11]. Moreover, high HVA levels in body fluids are associated with catecholamine-secreting tumors such as neuroblastoma, a cancer of the sympathetic nervous system that affects children under the age of five [12], or pheochromocytoma, a rare tumor of the adrenal medulla [13]. Elevated concentrations of HVA were also found in patients with autism [14], bulimia [15], schizophrenia [16], or post-traumatic stress disorder [17]. It is worth noting that HVA is a potential antioxidant [18].

Taking into account all the above, the importance of HVA monitoring in different biological fluids is obvious. A review of the literature shows that the most commonly used technique for HVA determination is HPLC with electrochemical detection [19,20], with the developed methods differing mostly in the mobile phase and electrochemical conditions. Instrumental methods based on gas chromatography–mass spectrometry [21], capillary electrophoresis [22], immunoassays [23], or fluorescence [24] have also been reported. HVA is widely employed as fluorometric substrate for oxidative enzymes [25].

Analytical methods based on electrochemical techniques are widely used for HVA determination and represent an alternative to those mentioned above since they do not require complex sample processing procedures and are reasonably priced. Among these, differential pulse voltammetry (DPV) and square wave voltammetry (SWV) are commonly applied for HVA quantification. Most of the sensors employed are carbon-based, modified or unmodified, and include glassy carbon [26,27,28,29,30,31,32], boron-doped diamond [30,33,34], screen printing [35,36,37,38], carbon paste [39,40,41], or graphite electrodes [42,43,44]. The main challenge with sensors based on different electrode materials is the need for surface regeneration and/or modification, which introduces additional steps in the sensor manufacturing process, as well as higher costs associated with the use of more reagents and specific procedures.

The present work exploits the advantages of the pencil graphite electrode (PGE) in order to study the electrochemical behavior, as well as the detection of HVA in human plasma. This type of electrode has recently been widely used in view of the various benefits, including high surface area and good conductivity, mechanical stability, favorable signal-to-noise ratio, affordability, and not last the possibility to determine a wide variety of analytes from different complex matrices. The strength of the present study consists of the combined advantages of the sensor: it is unmodified, disposable, and has similar or even better performance characteristics than some of the previously reported sensors.

## 2. Results and Discussion

### 2.1. Voltammetric Behavior of Homovanilic Acid at the Pencil Graphite Electrode

**Figure 1:**
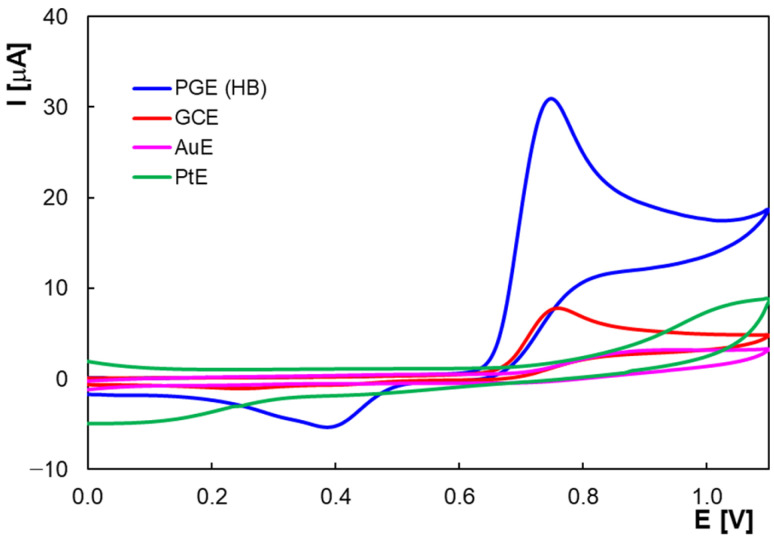
Cyclic voltammograms of 2.0 × 10^−4^ M HVA in BRB pH 2.0 using different electrodes; scan rate 0.100 V/s.

It is proven that the working electrode material is an important factor in the optimization of the electrochemical method of any analyte. In the present work, different types of materials were tested: gold, platinum, glassy carbon, and graphite (as PGE). Figure 1 shows the cyclic voltammograms for a 2.0 × 10^−4^ M HVA solution in BRB (pH 2.0) using these working electrodes. It can be noted that signals for HVA at the surface of the metal electrodes are not well defined. In contrast, using electrodes based on carbonaceous materials, an anodic peak at +0.75 V in BRB solution with pH 2.00 is observed, with the cyclic voltammogram shape indicating an irreversible electro-oxidation of HVA at these electrodes. As can be seen in the figure, the peak current measured at the PGE was at least three times higher than in the case of the GCE. However, in order to compare the performance of the electrodes, their sensitivities towards HVA, expressed as A × cm^−2^ × M^−1^, were calculated. The findings showed that the PGE (0.721 A × cm^−2^ × M^−1^) was 1.7 times more sensitive than the GCE (0.424 A × cm^−2^ × M^−1^).

**Figure 2:**
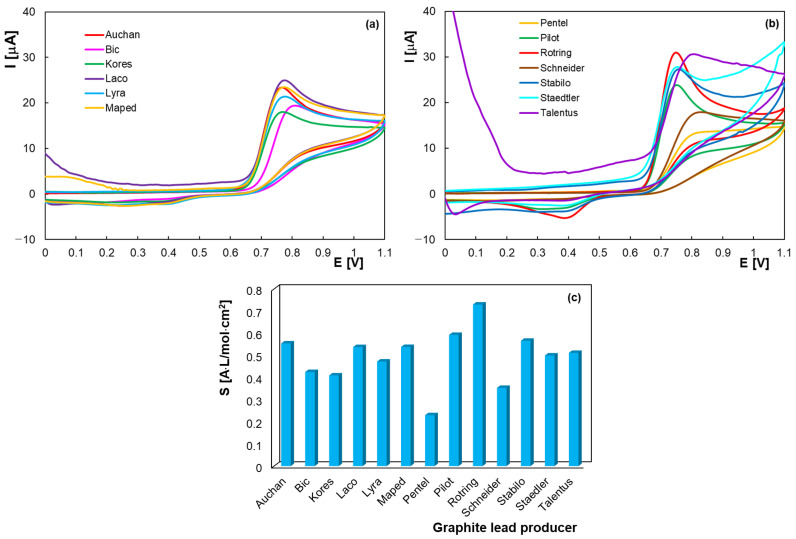
Cyclic voltammograms of 2.0 × 10^−4^ M HVA in BRB pH 2.0 at PGEs (HB) from different manufacturers (**a**,**b**); scan rate 0.100 V/s and sensitivities of HB leads from different manufacturers for HVA (**c**).

Another consideration for the selection of the working electrode material was the type of the graphite lead. HB-type graphite leads from several manufacturers were tested, with the cyclic voltammograms being recorded for HVA in BRB solution with pH 2.0 (Figure 2a,b). It should be noted that no measurable HVA redox signals were obtained for some types of the graphite leads, and in the other cases the peaks were more or less defined, with peak potentials that varied between +0.70 and +0.80 V. The sensitivities of the graphite-based electrodes to HVA were calculated as previously mentioned and are shown in Figure 2c. Sensitivities above 0.5 A × cm^−2^ × M^−1^ were obtained for the Rotring, Pilot, Stabilo, Auchan, Maped, Laco and Talentus leads, with the lowest value (below 0.3 A × cm^−2^ × M^−1^) being calculated for the Pentel leads. The differences observed in the HVA electrochemical behavior may be due to the influence of the graphite lead composition and also to the manufacturing process, about which there is little information.

**Figure 3:**
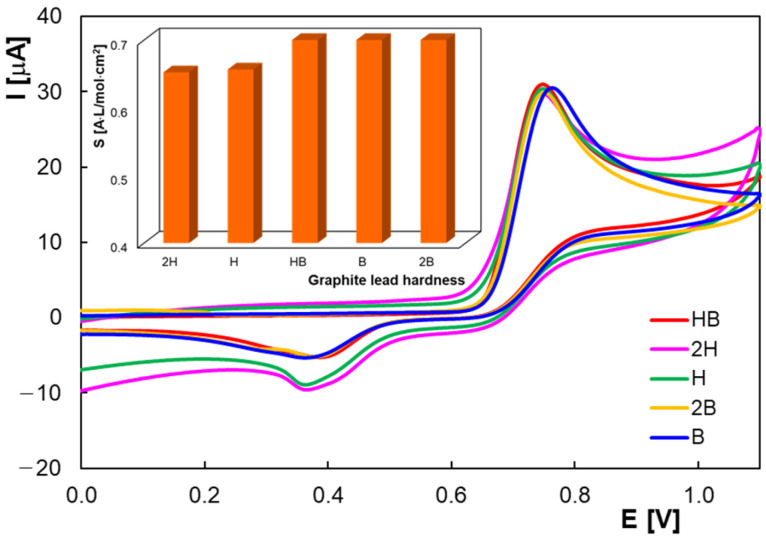
Cyclic voltammograms of 2.0 × 10^−4^ M HVA in BRB pH 2.0 at PGEs (Rotring) with different hardness; scan rate 0.100 V/s.

The composition of the graphite leads, more precisely their hardness, which depends on the ratio of graphite to binder, can also influence the electrochemical properties [45]. Rotring graphite leads of different hardness were selected for the study, and the cyclic voltammograms obtained for HVA are shown in Figure 3. Although relatively similar sensitivities were obtained for all the tested leads (inset of Figure 3), the HVA anodic peak was somewhat better defined at the HB-PGE. In addition, this type of lead is more readily available commercially and was therefore used in the subsequent investigations.

**Figure 4:**
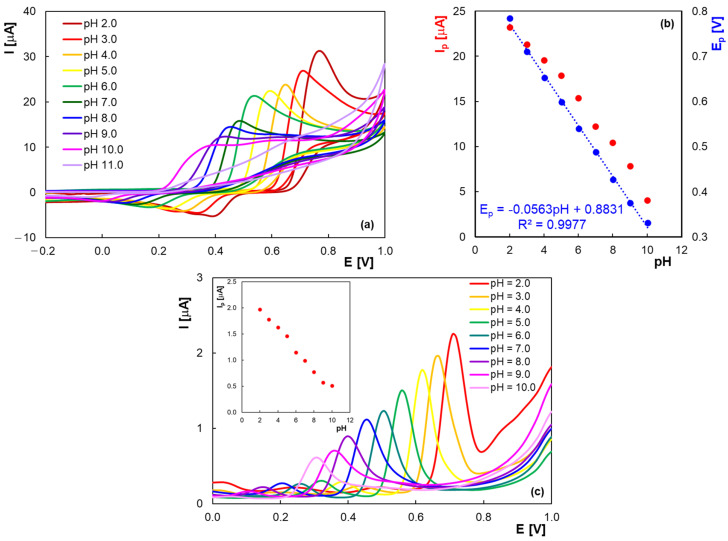
Cyclic voltammograms of 2.0 × 10^−4^ M HVA (**a**); dependence of the peak potential and the peak current on the pH (**b**) and differential pulse voltammograms of 2.0 × 10^−5^ M HVA (**c**) at PGE in BRB solutions with different pHs; scan rate 0.100 V/s.

The pH influence on the HVA electrochemical signal was studied in BRB with pH ranging from 2.0 to 11.0. As can be seen in Figure 4, at pH 2.0 an anodic peak due to HVA electro-oxidation appears at +0.75 V. On the reverse potential scan (Figure 4a), a poorly defined cathodic signal appears at about +0.4 V, probably due to the reduction in HVA oxidation compounds on the PGE surface. As the pH value of the supporting electrolyte solution increases, the potential of the HVA oxidation peak shifts to less positive values (approximately +0.3 V at pH 10.0), indicating the involvement of protons in the electrode process. At pH > 8.0, the oxidation peak is poorly defined. Additionally, a broad and very ill-defined second signal occurs at a 200 mV more positive potential. Moreover, at pH above 11, HVA is no longer electroactive at the PGE.

The slope (−0.056 V/pH) of the E_p_ vs. pH graph (Figure 4b) has a value close to the Nernstian one denoting that in the oxidation process of HVA, the number of protons involved in the electrode process is equal to that of electrons. In Figure 4b, one can observe that HVA oxidation peak current decreased with pH increasing, with similar results being obtained in DPV (Figure 4c). The best results in terms of sensitivity and the narrowest response were achieved at pH 2.0, and this value was therefore chosen for further experiments.

**Figure 5:**
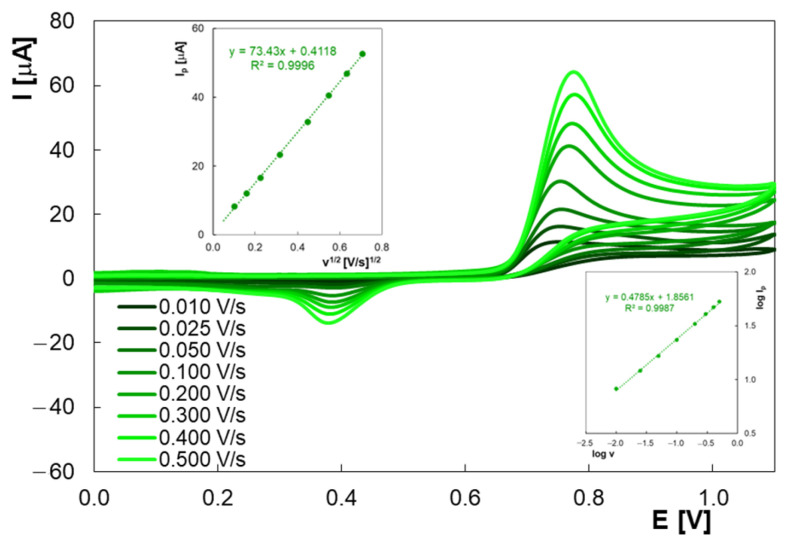
Cyclic voltammograms using PGE for 2.0 × 10^−4^ M HVA at different scan rates in BRB with pH 2.0; insets: I_p_ vs. v^1/2^ and log I_p_ vs. log v dependencies.

The scan rate (v) effect on HVA anodic peak was also studied. The experiments were performed at potential scan rates between 0.010 V/s and 0.500 V/s in BRB solution (pH 2.0) containing 2.0 × 10^−4^ M HVA (Figure 5). The anodic peak shifted towards positive potentials as the scan rate increased, indicating irreversible electro-oxidation of HVA. The oxidation peak current varied linearly with the square root of the scan rate (I_p_ (A) = 5.87 × 10^−5^ v^1/2^ (V × s^−1/2^) + 3.29 × 10^−7^, R^2^ = 0.9996), which means that the current is limited by HVA diffusion to the electrode surface. This finding was confirmed by the slope close to 0.5 of the log I_p_ vs. log v plot (inset of Figure 5), typical for a diffusion-controlled process.

The electron number involved in HVA electro-oxidation (*n*) was assessed applying the equation E_p_ − E_p/2_ = 47.7 mV/(1 − α) n, where α is the charge transfer coefficient and E_p/2_ is the potential measured at half of the peak height. Taking into consideration that most electrochemical systems are characterized by values of α between 0.3 and 0.7, and they can usually be approximated by 0.5 for new and unknown systems [46], n was calculated as 2. These outcomes show that HVA electro-oxidation involves the transfer of two electrons and two protons.

**Figure 6:**
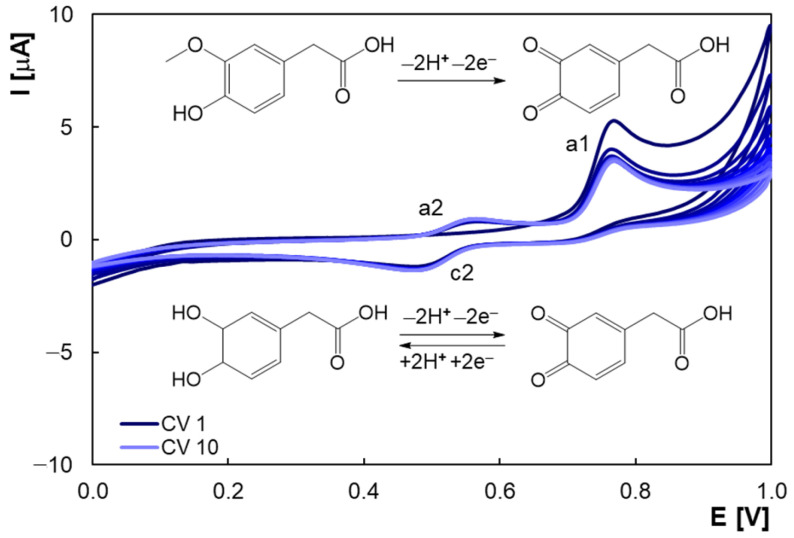
Ten successive cyclic voltammograms obtained using PGE for 2.0 × 10^−5^ M HVA in BRB solution with pH 2.0; scan rate 0.100 V/s.

According to the mechanism proposed by Mulla et al. [39], HVA oxidation occurs by removal of the methyl group to form 4-aceto-o-quinone (peak a1 in Figure 6). The formation of DOPAC is responsible for the cathodic signal (peak c2) in the reverse potential scan. In subsequent potential scans, DOPAC is oxidized (peak a2) to re-form 4-aceto-o-quinone, which is additionally generated by further HVA oxidation [39]. Figure 6 highlights that the decrease in the peak a1 current was approximately 20%, predicting electrode fouling that involves blocking HVA oxidation sites.

Given that the electrochemical oxidation of various phenolic compounds can involve dimerization or polymerization reactions [30] and also that HVA can form dimers in oxidation reactions [3,25,47], adsorption of the intermediates resulting from the reaction of phenoxy radicals with the parent compound can cause electrode passivation [30]. Some electrospray ionization mass spectrometry and fluorescence studies suggested that dimerization of HVA is unlikely [19]. Therefore, it can be said that further in-depth research is needed to elucidate the reaction mechanism.

### 2.2. Quantitative Determination of Homovanillic Acid by Differential Pulse Voltammetry

**Figure 7:**
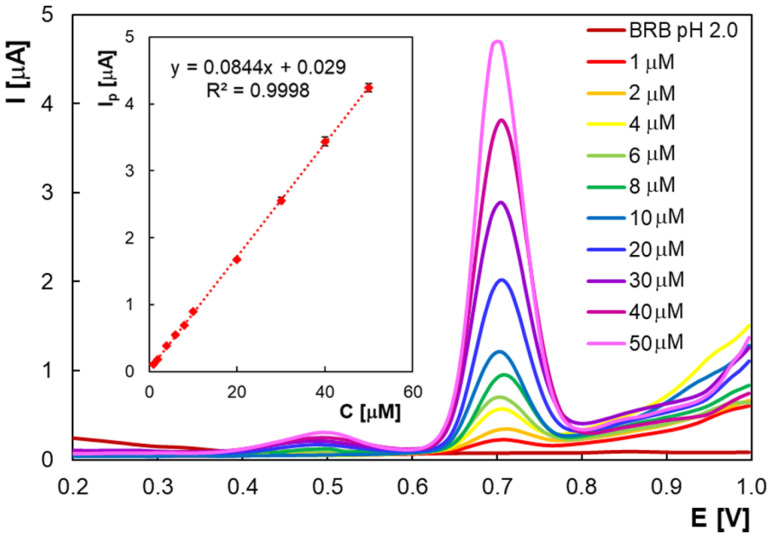
Differential pulse voltammograms recorded for different HVA concentrations in BRB (pH 2.0) and the corresponding calibration curve (inset).

In order to develop a voltammetric methodology for HVA determination, the DPV technique was used since it has better resolution and higher current sensitivity than CV. The performance parameters of the analytical method for HVA were evaluated starting with the calibration graph. In this regard, differential pulse voltammograms were recorded for solutions with concentrations of HVA between 1 × 10^−6^ M and 5 × 10^−5^ M (Figure 7) in BRB with pH 2.0. One can observe the appearance of a small additional peak at about +0.5 V which can be explained by the formation of intermediates in the HVA electro-oxidation mechanism (see Section 2.1). The relationship between HVA peak current at +0.7 V can be described by the equation I_p_ (A) = (0.0844 ± 0.0005) × C (M) + (3.0 ± 1.0) × 10^−8^ (R^2^ = 0.9988). The relative standard deviation (RSD%) calculated for the results of three replicates under repeatability conditions did not exceed 4.05%.

The detection limit (LOD = 3 × SD/m) of 3.84 × 10^−7^ M and the quantification limit (LOQ = 10 × SD/m) of 1.28 × 10^−6^ M were calculated using the standard deviation of the intercept (SD) and the slope of the calibration curve (m). The main performance characteristics of the proposed method are compared with those of other electroanalytical methods (Table 1). Two thirds of the studies presented in the table included modified electrodes, with the outcome being the increase in the time and cost of the analysis. For studies using unmodified electrodes, either surface regeneration is required [30,40] or electrochemical activation for 20 min [30,33,34]. The use of disposable SPCEs [35,37,38] is not financially advantageous compared to PGEs. Although most of the methods in Table 1 have quite close detection limits, the developed method has the advantage that it is designed to work with unmodified and disposable PGEs, which eliminates many of the steps required to clean and/or modify the electrode surface, providing shorter analysis time, higher sensitivity, and lower reagent consumption. Moreover, both the detection limit (3.84 × 10^−7^ M) and the linear range of the current method which uses the unmodified PGE (1.0 × 10^−6^ M − 5.0 × 10^−5^ M) were better than when the same type of PGE modified with PASA was employed (1.82 × 10^−6^ M and 3 × 10^−6^ M − 6.14 × 10^−5^ M, respectively) [43].

However, there are certain limitations regarding the applicability of the developed sensor and method, namely, real-time measurements for in vivo applications.

The accuracy and precision of the method were estimated intra-day and inter-day (three days) by analyzing three samples of HVA standard solution with different concentrations (low, medium, and high) prepared in triplicate, within the working range. Accuracy was expressed as relative error (r.e.% = 100 * (C_found_ − C_added_)/C_added_)) and precision was evaluated as percentage relative standard deviation (RSD%). The investigation results are presented in Table 2. The method was characterized by good precision (RSD% values that did not exceed 3.25%) and the calculated r.e.% was less than 2.92%, demonstrating good accuracy.

**Figure 8:**
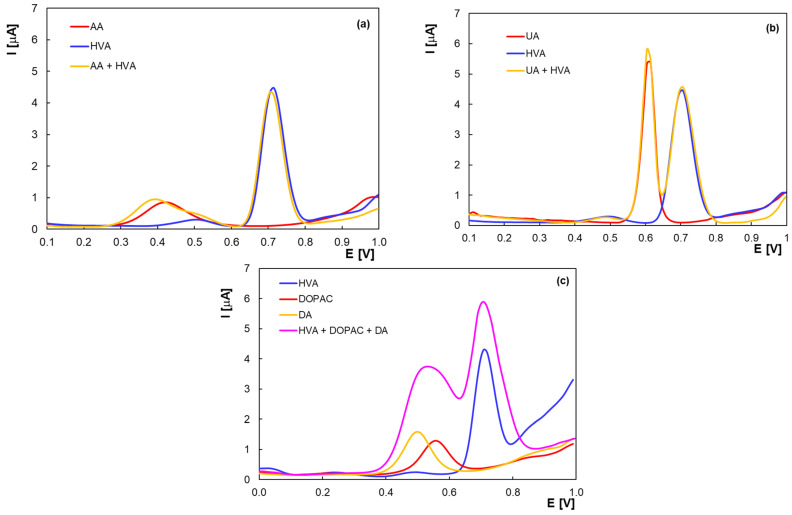
Differential pulse voltammograms for 5.0 × 10^−5^ M HVA in presence of (**a**) 5.0 × 10^−5^ M AA; (**b**) 5.0 × 10^−5^ M UA; and (**c**) 5.0 × 10^−5^ M DA and 5.0 × 10^−5^ M DOPAC in BRB with pH 2.0.

A major problem that hinders the electrochemical detection of the neurotransmitters and their metabolites is the interference of the compounds that coexist in biological matrices. Using the optimized DPV parameters, voltammetric curves of HVA were recorded in the presence of ascorbic acid (AA) and uric acid (UA), the main interfering compounds commonly found in biological samples (Figure 8a,b). The determination of HVA is feasible in the presence of these compounds because the signals corresponding to the electro-oxidation of AA (+0.4 V) and UA (+0.6 V) do not overlap with the HVA peak.

Another significant challenge is the determination of catecholamine neurotransmitters in the presence of their metabolites. Figure 8c shows the cyclic voltammograms obtained for individual solutions of HVA, DOPAC, and DA and also for a mixture of them in BRB with pH 2.0. Although it is not possible to determine the three compounds simultaneously because the DA and DOPAC peaks overlap, the analytical signal of HVA at about +0.7 V was not significantly affected by the presence of the other two compounds. The distinct signals and the difference of more than 100 mV between the peak potential of HVA and the peak potentials of the considered potential-interfering compounds prove the selectivity of the PGE towards HVA.

**Figure 9:**
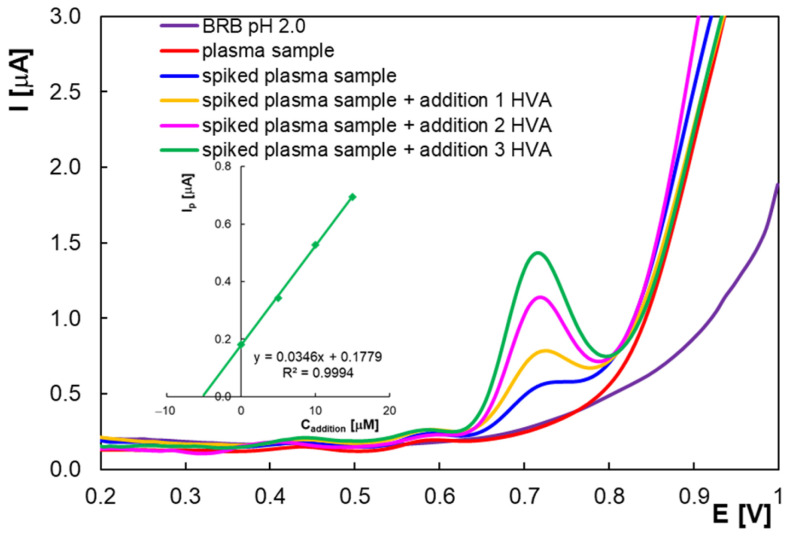
Differential pulse voltammograms obtained for a plasma replicate sample in BRB pH 2.0, spiked sample (5.0 × 10^−6^ M HVA), and spiked plasma after each of the three successive additions of 10 µL of 5.0 × 10^−3^ M HVA; calibration graph (inset).

The developed DPV method was applied for HVA analysis from spiked plasma samples (three replicate samples containing 5.0 × 10^−6^ M HVA). The HVA plasma concentrations ((5.15 ± 0.14) × 10^−6^ M) were determined using the standard addition method, with three different additions (0.01 mL) of 5.0 × 10^−3^ M HVA to the spiked plasma samples. The separated HVA signal was obtained, and the current increased proportionally to the concentration of HVA added (Figure 9—voltammograms obtained for one replicate sample). The recoveries were between 99.76% and 105.16%, and the average RSD% was 2.86%. The good recovery and the particularly good selectivity make the unmodified PGE a convenient tool for the determination of HVA from complex samples containing relevant interferences.

## 3. Materials and Methods

### 3.1. Chemicals and Samples

The analytical standard of HVA was acquired from Sigma Aldrich^®^ (St. Louis, MO, USA) and the corresponding mass was weighed and dissolved in double-distilled water to yield a 5 × 10^−3^ M stock solution. The freshly prepared stock solution was further diluted with supporting electrolytes to be subjected to the electrochemical measurements. The electrochemical behavior of HVA was investigated in 0.15 M Britton–Robinson buffer (BRB) solutions with pH values ranging from 2.0 to 11.0. The BRB solutions consisting of a mixture of acids (0.04 M H_3_PO_4_, H_3_BO_3_ and CH_3_COOH) and NaOH was used to adjust the pH. Potential interferents for HVA detection, including uric acid (UA), ascorbic acid (AA), DA, and DOPAC, were of analytical grade and were purchased from Sigma Aldrich^®^.

Human plasma samples (provided by a local hospital) were spiked with HVA and diluted with BRB pH 2.0, with the analyte concentration in the voltammetric cell being 5 × 10^−6^ M. The sample solution thus prepared was subjected to DPV measurement. Three such replicate samples were analyzed. For the quantitative HVA determination, the method of standard successive additions (3 additions of 10 µL each of 5 × 10^−3^ M HVA) was used.

### 3.2. Instrumentation

The experimental electrochemical configuration consisted of three electrodes connected to an AUTOLAB model PGSTAT 302N potentiostat (Metrohm, Barendrecht, The Netherlands), controlled by Nova 1.11 software. The three-electrode system included the working electrode, a platinum auxiliary electrode and an Ag/AgCl/Cl^–^ reference electrode. The tested working electrodes were a PGE (0.159 cm^2^ area), a glassy carbon electrode (GCE, 0.071 cm^2^ area), a platinum electrode (PtE, 0.031 cm^2^ area), and a gold electrode (AuE, 0.031 cm^2^ area). In addition, 0.5 mm graphite leads of various hardnesses (H, 2H, HB, B, 2B) from Rotring and HB graphite leads from different manufacturers were examined. Graphite leads of 6 cm length were cut in half and the cut part was inserted into the mechanical pencil used as a holder. A copper wire welded to the metal part of the pencil provided the electrical contact. To ensure a reproducible electroactive surface area of the electrode, 1 cm of the graphite lead was immersed in the solution to be analyzed [51]. The volume of all solutions in the electrochemical cell was 10 mL, and a new graphite lead was used for each experiment. Five replicate samples were prepared for each solution. The GCE, PtE, and AuE were polished with alumina powder (0.05 µm) and then washed with distilled water and dried.

### 3.3. Electrochemical Measurements

In the investigations, cyclic voltammetry (CV) and PGE were employed to study HVA electrochemical behavior at various scan rates and pH values. Optimized HVA detection was achieved through the use of DPV and PGE HB-type. The CV scans were performed from −0.2 V to +1.1 V (vs. Ag/AgCl/Cl^–^). DPV measurements carried out under the following conditions: potential window between +0.2 V and +1.0 V, step potential 5 mV, pulse length 25 mV, pulse time 0.05 s, time interval between pulses 0.5 s.

The pH of the supporting electrolytes was measured using a Consort C6010 pH-meter (Fisher Scientific, Merelbeke, Belgium). All the experiments were performed at 25 °C.

## 4. Conclusions

The present study included the investigation of HVA oxidation using electrochemical techniques and a PGE as working electrode. HVA was irreversibly oxidized at the PGE in BRB solutions, with the best-defined signal being obtained in solution with pH 2.0. Under these conditions, the HVA electro-oxidation was diffusion-controlled, involving two electrons and two protons.

Direct determination of HVA was performed using DPV and a linear relationship between the peak current and HVA concentration was obtained. Both the linear range and the detection limit were comparable or even better than those previously reported. Also, the proposed method was accurate, precise, and rapid enough to be used in the HVA routine analysis. Another strength of the proposed method is the possibility of determining HVA in the presence of important compounds that are found in complex biological samples, such as UA and AA, but especially along with dopamine and another of its metabolites, namely DOPAC. These proven advantages, as well as the satisfactory results obtained in the analysis of plasma samples, demonstrate that the PGE is a good and attractive candidate for practical applications.

The results indicated that the presented DPV method and PGE are a potential electroanalytical alternative and a satisfactory substitute for the methods that involve separations due to being much faster and cheaper than other methods for HVA quantification as an index of brain dopamine.

## Figures and Tables

**Table 1:** Overview of electroanalytical methods for HVA determination.

Electrode	Technique	Supporting Electrolyte	Linear Range [μM]	Detection Limit [μM]	Sample	Ref
MIP/GCE	DPV	0.1 M citrate/HCl (40% acetonitrile) (pH 1.1)	0.05–10	0.007	–	[26]
Poly-AMT/GCE	Amp	0.2 M PB (pH 7.2)	0.01–100	0.000094	plasma	[27]
Poly-ARS/GCE	DPV	0.1 M PB (pH 3.0)	0.09–160	0.017	plasma	[28]
ERC60–GO–Ph/GCE	SWV	PB (pH 7.0)	0.1–7.2	0.03	urine	[29]
Nafion/GCE	DPV	0.1 M PB (pH 3.0)	2–10 10–100	0.8	urine	[30]
GCE	DPV	0.1 M PB (pH 3.0)	2–10 10–100	0.9	urine	[30]
PNR/GCE	DPV	0.1 M PB (pH 3.0)	4–100	1.2	urine	[30]
PVB/GO/GCE	DPV	PB (pH 6.0)	0.549–120	0.18	urine	[31]
MWCNT-PtNP/GCE	DPV	0.1 M PB (pH 7.0)	0.2–80	0.08	urine	[32]
BDDE	DPV	0.1 M PB (pH 3.0)	2–100	0.6	urine	[30]
BDDE	FIA-AD	0.04 M BRB (pH 3.0)	1–10 10–100	0.44	-	[33]
BDDE	DPV	0.1 M PB (pH 6.0)	1.2–100	0.4	urine	[34]
SPCE	DPV	0.04 M BRB (pH 3.0)	0.2–100	0.24	urine	[35]
Poly-CTB/G-SPE	DPV	PB (pH 7.0)	40–100	2.2	artificial urine	[36]
SPCE	FIA-AD	0.04 M BRB (pH 2.0)	0.05–100	0.065	-	[37]
SPCE	DPV	BRB (pH 9.5)	0.25–2.5	0.4	urine	[38]
PEA/CPE	CV	PB (pH 7.4)	10–100	3	brain extracellular fluid	[39]
GCPE	DPV	BRB (pH 2.0)	1–100	1.26 *	-	[40]
ZFO-MWCNT/CPE	DPV	BRB (pH 2.0)	0.415–3.225	0.148	urine	[41]
Ni-ZnO NPs/CPE	DPV		3.96–0.383	1.01	urine	[48]
Poly-CB-oPD/GE	DPV	0.1 M PB (pH 7.2)	9.99–1220	-	-	[42]
Poly-ASA/PGE	DPV	PB (pH 8.0)	3–61.4	1.82	urine	[43]
Urea-derivative/GE	DPV	0.1 M PB (pH 6.0)	9.96–624	-	-	[44]
L-leu-Sol-Gel/CE	DPV	PB (pH 4.0)	0.4–100	0.1	urine	[3]
CCFE	DPV	BRB (pH 2.0)	0.8–100	0.3	-	[49]
MIP-CNT fiber	DPV	PB (pH 7.4)	0.01–2	0.00458	rat caudal vein	[50]
PGE	DPV	BRB (pH 2.0)	1–50	0.384	human plasma	this work

BDDE: boron-doped diamond electrode, GCE: glassy carbon electrode, MWCNT: multi-walled carbon nanotubes, PtNP: platinum nanoparticle, PB: phosphate buffer, DPV: differential pulse voltammetry, FIA-AD: flow injection analysis with amperometric detection, BRB: Britton–Robinson buffer, SPCE: screen-printed carbon electrode, CCFE: carbon composite film electrode, ASA: 4-aminosalicylic acid, PGE: pencil graphite electrode, ZFO: zinc ferrite nanostructure, CPE: carbon paste electrode, PEA: phosphatidylethanolamine, GCPE: glassy carbon paste electrode, PVB: polyvinyl butyral, GO: graphene oxide, ARS: Alizarin Red S, CTB: coumarin derivative of Tröger’s base, G-SPE: screen-printed electrode with graphite, L-leu-Sol-Gel/CE: L-leucine modified sol–gel carbon electrode, Ni-ZnO NPs: nickel-doped zinc oxide nanoparticles; ERC60–GO–Ph: phenyl modified fullerene–graphene oxide interface, CB-oPD: cobalt bis(dicarbollide) derivative that includes the o-phenylenediamine unit, GE: graphite electrode, AMT: 3-amino-5-mercapto-1,2,4-triazole, MIP: molecularly imprinted polymer. * Quantification limit.

**Table 2:** Accuracy and precision data of DPV method for HVA quantification; SD—standard deviation.

C_added_ [M]	Intra-Day			Inter-Day		
	C_recovered_ ± SD [M]	r.e.%	RSD%	C_recovered_ ± SD [M]	r.e.%	RSD%
1.00 × 10^−6^	1.00 × 10^−6^ ± 2.54 × 10^−8^	0.49	2.53	1.01 × 10^−6^ ± 3.33 × 10^−8^	3.31	3.25
1.00 × 10^−5^	1.03 × 10^−5^ ± 1.78 × 10^−7^	2.80	1.73	1.03 × 10^−5^ ± 1.86 × 10^−7^	2.92	1.80
5.00 × 10^−5^	4.99 × 10^−5^ ± 7.56 × 10^−6^	−0.21	1.52	4.95 × 10^−5^ ± 1.44 × 10^−6^	−0.14	2.92

## Data Availability

Data are contained within the article.
